# Interaction of Operator−Patient Sex on Peri‐Procedural Treatments and Major Coronary Outcomes After Percutaneous Coronary Intervention

**DOI:** 10.1002/ccd.70799

**Published:** 2026-08-05

**Authors:** Sharon A. Ayayo, Vicky P. Taxiarchi, Angela Jerath, Evangelos Kontopantelis, Sonya N. Burgess, Sarah Zaman, Mamas A. Mamas

**Affiliations:** ^1^ Faculty of Biology, Medicine and Health, Division of Informatics, Imaging and Data Sciences The University of Manchester Manchester UK; ^2^ Nuffield Department of Primary Care Health Sciences University of Oxford Oxford UK; ^3^ Faculty of Biology, Medicine and Health, Centre for Women's Mental Health, Division of Psychology and Mental Health The University of Manchester Manchester UK; ^4^ Department of Anesthesiology and Pain Medicine Sunnybrook Health Sciences Center Toronto Ontario Canada; ^5^ Institute for Health Policy, Management and Evaluation University of Toronto Toronto Ontario Canada; ^6^ ICES Toronto Ontario Canada; ^7^ Schulich Heart Centre, Sunnybrook Research Institute Sunnybrook Health Sciences Center Toronto Ontario Canada; ^8^ Division of Family Medicine, Yong Loo Lin School of Medicine National University of Singapore Singapore; ^9^ Centre for Research in Health Systems Performance National University of Singapore Singapore; ^10^ Cardiology Department University of Sydney and Sydney Southwest Hospital Sydney Australia; ^11^ Westmead Applied Research Centre, Faculty of Medicine and Health University of Sydney Sydney New South Wales Australia; ^12^ Department of Cardiology Westmead Hospital Sydney New South Wales Australia; ^13^ Keele Cardiovascular Research Group Keele University Stoke on Trent UK; ^14^ National Institute for Health and Care Research (NIHR) Birmingham Biomedical Research Centre Birmingham UK

**Keywords:** MACCE, Mortality, percutaneous coronary intervention, sex concordance, sex discordance

## Abstract

**Background:**

There are limited data evaluating the association between operator−patient sex, and outcomes in patients undergoing percutaneous coronary intervention (PCI).

**Methods:**

We included 138,112 consecutive PCI procedures in patients presenting with acute coronary syndrome (ACS) in England and Wales, 2017−2020. Primary endpoints of in‐hospital major adverse cardiovascular and cerebrovascular events (MACCE), and mortality were assessed. Risk ratios (RR) estimated independent associations of patient sex and patient−operator sex with peri‐procedural treatment and outcomes.

**Results:**

Female patients had a 10% higher risk of MACCE [95% CI: 1.04, 1.16], and a 14% higher risk of in‐hospital mortality than male patients [95% CI: 1.07, 1.21]. Male operators undertook the vast majority of PCI procedures; 130,604 (94.6%). Patient sex‐based differences in outcomes were not present for female operators but were statistically significant amongst male operators. Risk‐adjusted data found that, when treated by male operators, female patients experienced higher MACCE and mortality rates than male patients (4.1% vs. 3.7%; RR: 1.11 [95% CI: 1.05,1.18] and 3.1% vs. 2.7%; RR: 1.15 [95% CI: 1.08, 1.22] respectively). When treated by female operators, female patients experienced numerically lower MACCE rates than male patients (4.0% vs. 4.3%; RR:0.93 [95% CI: 0.72, 1.14] and similar mortality rates (2.9% vs. 2.9%; RR:1.01 [95% CI: 0.75, 1.26]). Operator−patient sex discordance was associated with lower transradial PCI (RR: 0.96 [95% CI: 0.95, 0.96]), lower prasugrel use (RR: 0.88 [95% CI: 0.82, 0.94]), higher MACCE (RR: 1.12 [95% CI: 1.06, 1.18]) and mortality (RR: 1.14 [95% CI: 1.07,1.21]) compared with operator−patient sex concordance.

**Conclusions:**

Female patients experienced higher risk‐adjusted mortality than male patients; while outcomes appeared less favorable among those treated by male operators, this finding should be interpreted cautiously given the limited precision for female operators.

**Abbreviations**
ACS: acute coronary syndrome
BCIS: British Cardiovascular Interventions Society
BMI: body mass index
CABG: coronary artery bypass grafting
CTO: chronic total occlusion
LVEF: left ventricular ejection fraction
MACCE: major adverse cardiovascular and cerebrovascular events
MI: myocardial infarction
MVL: multivessel
NHS: National Health Service
PCI: percutaneous coronary intervention
PVD: peripheral vascular disease
RR: risk ratio
STEMI: ST‐elevated myocardial infarction
UK: United Kingdom
VHD: valvular heart disease

## 1 Introduction

Percutaneous coronary intervention (PCI) is the most common means of coronary revascularization in patients with ACS. Despite advancements in PCI techniques, there remain differences in clinical outcomes between male and female patients undergoing PCI for ACS. Several past studies found women at greater risk of periprocedural complications with worse clinical outcomes [1, 2, 3, 4]. Female patients are often underrepresented in clinical trials and are less likely to receive guideline‐recommended quality of care compared with males [5, 6, 7, 8].

There are limited and contradicting data on the association between operator sex and patient outcomes, with some studies reporting better patient outcomes for those treated by female physicians [9, 10, 11], while some studies found no significant associations between physician sex and patient outcomes [12, 13, 14, 15]. A recent systematic review found that patients under the care of female physicians had significantly lower odds of mortality and experienced fewer hospital readmissions across both medical and surgical settings [9]. In the PCI setting, Yelavarthy et al. [12] found no significant differences between female and male interventional cardiologists in the risk‐adjusted rates of mortality, acute kidney injury, transfusion, or major bleeding in Michigan between 2010 and 2017. However, the applicability of such data to populations outside of North America is unclear, or in healthcare systems characterized by higher volume of operators or in publicly funded healthcare systems. Evidence from other clinical settings suggests that patient‐physician sex concordance may influence clinical decision‐making and outcomes, although findings have been heterogeneous across studies [16, 17, 18]. Furthermore, there is limited data on the association between the sex of the PCI operator and guideline‐recommended care like transradial PCI, intravascular imaging, and prescribing of potent antiplatelet therapy.

Therefore, the aim of this study was to explore the associations between operator and patients' sex on PCI quality indicators, as well as patient outcomes, using the national PCI registry in England and Wales. By understanding these dynamics, we can better inform clinical practices and potentially improve the quality of care for all patients undergoing PCI.

## 2 Methods

### 2.1 Study Design

Retrospective study of prospectively collected data from the British Cardiovascular Interventions Society (BCIS) database.

### 2.2 The BCIS Registry

This is a UK national registry that collects records of almost every PCI in a public hospital, including patient demographics, clinical presentation, pharmacological, procedural and in‐hospital outcomes [19]. Secondary use of anonymized data for research purposes is authorized under NHS research governance arrangements and further supported under section 251 of the NHS Act 2006 (NIGB: ECC1‐06(d)/2011), which allows researchers to use patient information collected within the dataset for medical research without patient consent.

### 2.3 Study Population and Inclusion/Exclusion Criteria

All PCI procedures performed on National Health Service (NHS) patients aged 18 to 100 years and admitted with ACS in England and Wales in 2017−2020. Patient‐level PCI data from Scotland and Northern Ireland were not included as these are not routinely submitted to the BCIS registry. Patients with stable angina, missing record of operator in charge, missing operator or patient sex, and missing in‐hospital outcomes were also excluded (Supporting Information S1: Figure 1). We restricted the analysis to ACS PCI to minimize heterogeneity related to case selection and clinical indication, as treatment pathways in this setting are more standardized than in elective PCI. The BCIS database only provides the operator's name; therefore, the operator's sex was determined through online searches and verified by knowledge within the research team.

### 2.4 Outcomes and Covariates

The outcomes of interest included guideline‐recommended peri‐procedural treatments: transradial PCI, intravascular imaging and newer potent antiplatelets use (Prasugrel and Ticagrelor). In‐hospital primary endpoints included mortality and MACCE, a composite endpoint of any Q‐wave or non‐Q wave MI, re‐infarction, re‐intervention, side branch occlusion, stroke and death. In‐hospital mortality was defined as any death that occurred after PCI and before discharge. Q‐wave MI was defined as creatine phosphokinase > x2 upper limit of normal with elevated creatine kinase‐MB Isoenzyme or Troponin and new pathological Q waves or new left bundle branch block (LBBB). Non‐Q wave was defined similarly except with no new pathological Q waves and no new LBBB.

Covariates were a‐priori determined to be clinically relevant and available in the dataset: age, ethnicity, smoking status, body mass index (BMI), previous MI, previous PCI, previous coronary artery bypass grafting (CABG), hypercholesterolemia, hypertension, peripheral vascular disease (PVD), previous stroke, vascular heart disease (VHD), left ventricular ejection fraction (LVEF), diabetes mellitus, kidney disease, multivessel disease, left main disease, chronic total occlusion (CTO), cardiogenic shock and PCI year.

### 2.5 Statistical Analysis

Descriptive statistics were stratified into four groups by operator and patient sex. Continuous variables were summarized by means (standard deviations) or median (interquartile ranges) as appropriate, and categorical variables were summarized by frequencies (percentages). Missing data on all covariates were summarized by numbers (percentages) to explore the level of incompleteness (Supporting Information S1: Table 1) and were subsequently imputed using multiple imputation by chained equations (MICE). Ten imputed data were produced with estimates pooled using Rubin's rule [20]. All covariates and outcomes were included in the imputation model, as generally recommended [21].

Chi‐square tests were used to explore associations between operator sex and each outcome separately for male, female, or all patients. Separate logistic regression models for each outcome examined for independent associations between patient sex and study outcomes, adjusted for all covariates. Additional multivariable logistic regression models included an interaction term of patient and operator sex. Risk ratios with 95% confidence intervals describe differences in patient sex‐based outcomes by operator sex. Lastly, differences in outcomes by patient‐operator sex concordance or discordance were examined through additional multivariable logistic regression models per outcome. Concordance refers to PCIs were male operators treated male patients or female operators treated female patients, and discordance to PCIs were male operators treated female patients or female operators treated male patients.

A subgroup analysis of all outcomes was implemented for patients with STEMI only, with the same statistical methods.

Analyses were performed using Stata/MP v18 [22]. All p‐values were 2‐sided, and a 5% level of significance was used.

## 3 Results

The study included 138,112 PCI procedures performed in England and Wales, 2017‐2020. There were 928 operators, 872 (94.0%) were males and 56 (6.0%) females. The majority of PCIs were performed by male operators performed 130,604 (94.6%) PCIs. Male patients accounted for 101,260 (73.3%) procedures and female patients for 36,852 (26.7%). Sex concordance was met for 97,742 (70.8%) procedures (majorly for males treating males: 98%), and sex discordance for 40,370(29.2%) (majorly for males treating females: 86.3%) (Table 1). Male patients were generally younger than females but more likely to have comorbidities including previous MI, CABG or PCI, and kidney disease. Males also presented more complex disease, including multivessel disease, left main disease and CTO, whereas females were more likely to have hypertension, diabetes mellitus, previous stroke, history of VHD and cardiogenic shock. Patients treated by male operators were more likely to have previous CABG, hypercholesterolemia, hypertension and multivessel disease, while those treated by female operators were more likely to have previous stroke, VHD and poor/moderate LVEF. Female operators were also more likely to treat non‐white patients (Table 1).

**Table 1:** Demographics and clinical characteristics tabulated by sex of patients and operators (*n*, [%]).

Operator sex	Male			Female			Overall
Patient sex	Male	Female	Total	Male	Female	Total	
*N*	95,747	34,857	130,604	5513	1995	7508	138,112
Age, mean (SD)	63.8 (12.1)	68.8 (12.3)	65.2 (12.4)	63.0 (12.0)	68.4 (12.4)	64.5 (12.4)	65.1 (12.4)
Ethnicity							
White	62,946 (82.9)	23,609 (85.4)	86,555 (83.6%)	3694 (79.0)	1393 (80.9)	5087 (79.5%)	91,642 (83.3)
Not White	12,982 (17.1)	4051 (14.6)	17,033 (16.4%)	983 (21.0)	328 (19.1)	1311 (20.5%)	18,344 (16.7)
BMI, mean (SD)	28.3 (5.0)	28.3 (6.2)	28.3 (5.4)	28.0 (5.0)	28.9 (6.2)	28.5 (5.3)	28.3 (5.4)
*Smoking*							
Never smoker	32,124 (36.4)	15,416 (48.3)	47,540 (39.5%)	1901 (36.8)	912 (49.4)	2813 (40.1%)	50,353 (39.6)
Current/Ex smoker	56,143 (63.6)	16,534 (51.7)	72,677 (60.5%)	3765 (63.2)	935 (50.6)	4200 (59.9%)	76,877 (60.4)
*Previous MI*							
No	72,986 (77.4)	27,769 (80.8)	100,755 (78.3%)	4166 (76.5)	1621 (82.3)	5787 (78.0%)	106,542 (78.3)
Yes	21,338 (22.6)	6598 (19.2)	27,936 (21.7%)	1279 (23.5)	349 (17.7)	1628 (22.0%)	29,564 (21.7)
*Previous CABG*							
No	89,114 (93.7)	33,391 (96.4)	122,505 (94.5%)	5169 (94.6)	1916 (96.9)	7085 (95.2%)	129,590 (94.5)
Yes	5955 (6.3)	1241 (3.6)	7196 (5.5%)	297 (5.4)	61 (3.1)	358 (4.8%)	7554 (5.5)
*Previous PCI*							
No	75,317 (79.5)	28,511 (82.6)	103,828 (80.3%)	4307 (79.0)	1644 (83.7)	5951 (80.3%)	109,779 (80.3)
Yes	19,406 (20.5)	5996 (17.4)	25402 (19.7%)	1143 (21.0)	321 (16.3)	1464 (19.7%)	26,866 (19.7)
*Hypercholesterolemia*							
No	52,730 (56.1)	18,813 (54.8)	71,543 (55.8%)	3073 (57.0)	1131 (57.6)	4204 (57.2%)	75,747 (55.8)
Yes	41,295 (43.9)	15,486 (45.2)	56,781 (44.2%)	2319 (43.0)	833 (42.4)	3152 (42.8%)	59,933 (44.2)
*Hypertension*							
No	45,963 (48.9)	14,350 (41.8)	60,313 (47.0%)	2678 (49.7)	841 (42.8)	3519 (47.8%)	63,832 (47.0)
Yes	48,062 (51.1)	19,949 (58.2)	68,011 (53.0%)	2714 (50.3)	1123 (57.2)	3837 (52.2%)	71,848 (53.0)
*PVD*							
No	90,333 (96.1)	33,064 (96.4)	123,397 (96.2%)	5187 (96.2)	1885 (96.0)	7072 (96.1%)	130,469 (96.2)
Yes	3692 (3.9)	1235 (3.6)	4927 (3.8%)	205 (3.8)	79 (4.0)	284 (3.9%)	5211 (3.8)
*Previous stroke*							
No	90,300 (96.0)	32,624 (95.1)	122,924 (95.8%)	5144 (95.4)	1853 (94.3)	6997 (95.1%)	129,921 (95.8)
Yes	3725 (4.0)	1675 (4.9)	5400 (4.2%)	248 (4.6)	111 (5.7)	359 (4.9%)	5759 (4.2)
*Valvular heart disease*							
No	92,466 (98.3)	33,478 (97.6)	125,944 (98.1%)	5280 (97.9)	1914 (97.5)	7194 (97.8%)	133,138 (98.1)
Yes	1559 (1.7)	821 (2.4)	2380 (1.9%)	112 (2.1)	50 (2.5)	162 (2.2%)	2542 (1.9)
*LVEF*							
Good	26,465 (57.4)	10,324 (60.9)	36,789 (58.4%)	1327 (55.5)	463 (55.6)	1790 (55.5%)	38,579 (58.2)
Moderate/poor	19,616 (42.6)	6637 (39.1)	26,253 (41.6%)	1065 (44.5)	369 (44.4)	1434 (44.5%)	27,687 (41.8)
*Diabetes mellitus*							
No	72,862 (77.4)	25,338 (73.9)	98,200 (76.4%)	4141 (76.6)	1461 (74.5)	5602 (76.1%)	103,802 (76.4)
Yes	21330 (22.6)	8942 (26.1)	30272 (23.6%)	1263 (23.4)	501 (25.5)	1764 (23.9%)	32036 (23.6)
*Kidney disease*							
No	90,050 (97.2)	32,855 (97.3)	122,905 (97.2%)	5231 (97.1)	1902 (97.8)	7133 (97.3%)	130,038 (97.2)
Yes	2630 (2.8)	926 (2.7)	3556 (2.8%)	157 (2.9)	43 (2.2)	200 (2.7%)	3756 (2.8)
*Cardiogenic shock*							
No	91,414 (96.1)	33,149 (95.8)	124,563 (96.1%)	5278 (96.5)	1908 (96.0)	7186 (96.3%)	131,749 (96.1)
Yes	3671 (3.9)	1439 (4.2)	5110 (3.9%)	194 (3.5)	79 (4.0)	273 (3.7%)	5383 (3.9)
*MVL disease*							
No	61,501 (67.9)	23,931 (72.5)	85,432 (69.1%)	3746 (72.4)	1392 (74.7)	5138 (73.0%)	90,570 (69.3)
Yes	29,064 (32.1)	9088 (27.5)	38,152 (30.9%)	1427 (27.6)	472 (25.3)	1899 (27.0%)	40,051 (30.7)
*Left main disease*							
No	77,736 (96.0)	28,560 (96.5)	106,296 (96.1%)	4125 (96.1)	1503 (96.5)	5628 (96.2%)	111,924 (96.1)
Yes	3234 (4.0)	1026 (3.5)	4260 (3.9%)	167 (3.9)	55 (3.5)	222 (3.8%)	4482 (3.9)
*CTO*							
No	89,098 (96.5)	32,754 (97.5)	121,852 (96.8%)	5170 (96.7)	1878 (97.6)	7048 (97.0%)	128,900 (96.8)
Yes	3184 (3.5)	830 (2.5)	4014 (3.2%)	174 (3.3)	46 (2.4)	220 (3.0%)	4234 (3.2)

Abbreviations: BMI, body mass index; CABG, coronary artery bypass grafting; CTO, chronic total occlusion; LVEF, left ventricular ejection fraction; MACCE, major adverse cardiovascular and cerebrovascular events; MI, myocardial infarction; MVL, multivessel; PCI, percutaneous coronary intervention; PVD, peripheral vascular disease.

Unadjusted estimates of the outcomes indicate that, overall, male operators performed a higher proportion of transradial PCIs compared to female operators (88.3% vs. 86.8%; *p* < 0.001), whereas female operators were more likely to prescribe ticagrelor than male operators (52.8% vs. 50.6%; *p* = 0.001), and the difference was more profound in male patients (Figure 1). Before adjustment for confounders, it was observed that male patients treated by female operators experienced a higher rate of MACCE compared to those treated by male operators (4.1% vs. 3.6%; *p* = 0.036), and no other statistically significant differences were indicated between operator‐patient sex and peri‐procedural outcomes or in‐hospital mortality.

**Figure 1:**
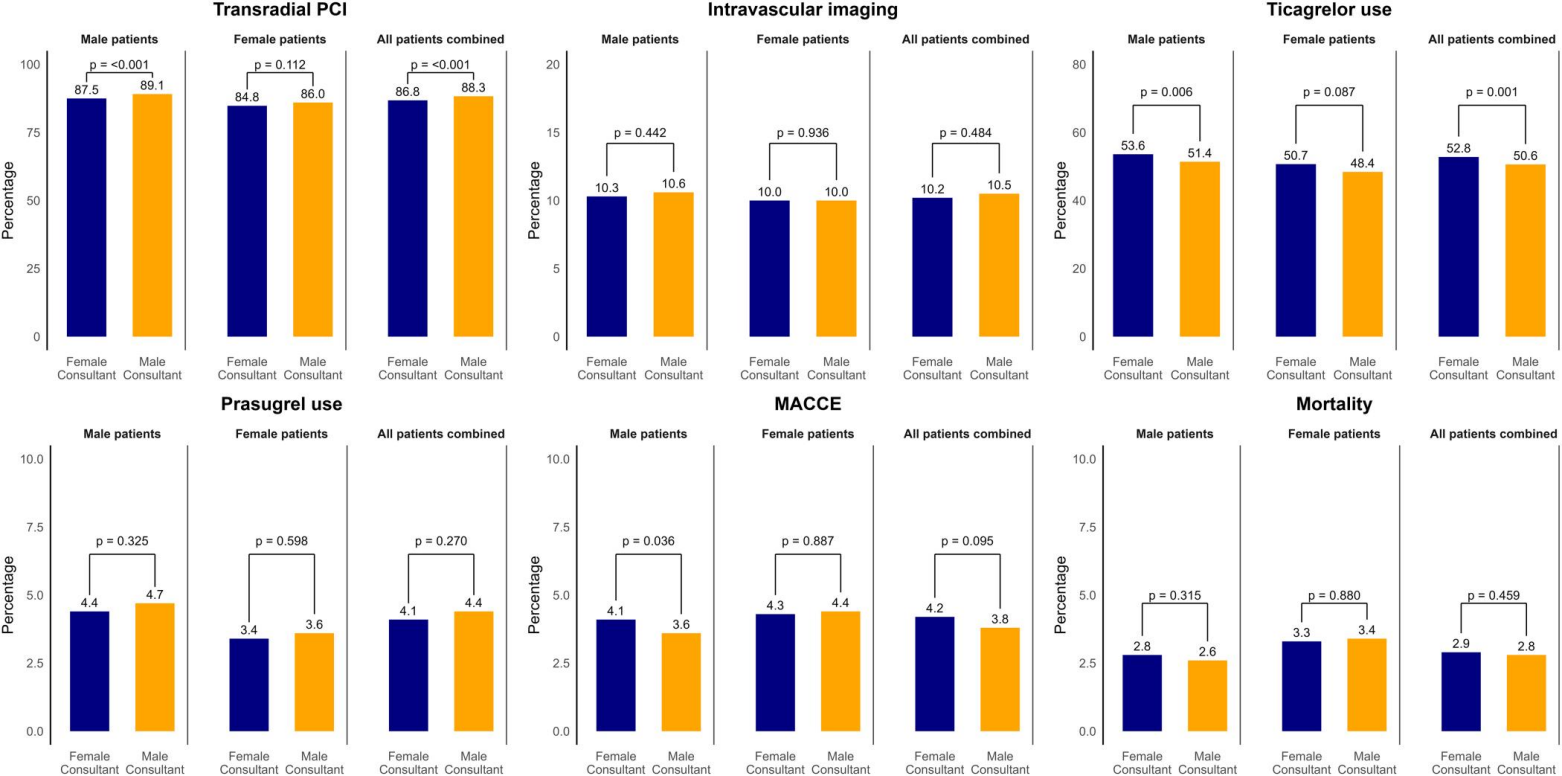
Observed (unadjusted) periprocedural and adverse hospital outcomes by operator–patient sex. MACCE, Major Adverse Cardiovascular and Cerebrovascular Events; PCI, Percutaneous Coronary Intervention. [Color figure can be viewed at wileyonlinelibrary.com]

However, following adjustment for covariates and across all operators, female patients were statistically significantly less likely than male patients to undergo a transradial PCI procedure (RR: 0.95 [95% CI: 0.95,0.96]), receive prasugrel (RR: 0.87 [95% CI: 0.81, 0.93]), or ticagrelor (RR: 0.98 [95% CI: 0.97, 1.00]) (Table 2). There was no indication that these findings differed after stratification by operator's sex (Figure 2). Patient−operator sex discordance was associated with less prescribing of prasugrel (RR: 0.88 [95% CI: 0.82, 0.94]) and less transradial PCI (RR: 0.96 [95% CI: 0.95, 0.96]) compared to operator−patient sex concordance (Table 3).

**Table 2:** Independent association of patient's gender with periprocedural treatments and adverse hospital outcomes.

	Female patient % (*N* = 36,852)	Male patient % (*N* = 101,260)	RR (95% CI)	*p* value
Transradial PCI	85.0	89.3	0.95 (0.95, 0.96)	<0.001
Intravascular Imaging	10.4	10.4	1.00 (0.97, 1.04)	0.941
Prasugrel use	4.1	4.7	0.87 (0.81, 0.93)	<0.001
Ticagrelor use	50.2	51.1	0.98 (0.97, 1.00)	0.016
MACCE	4.1	3.7	1.10 (1.04, 1.16)	0.001
Mortality	3.1	2.7	1.14 (1.07, 1.21)	<0.001

Abbreviations: MACCE, major adverse cardiovascular and cerebrovascular events; PCI, percutaneous coronary intervention.

Estimates extracted following adjustment for covariates; ref. = Male patient.

**Figure 2:**
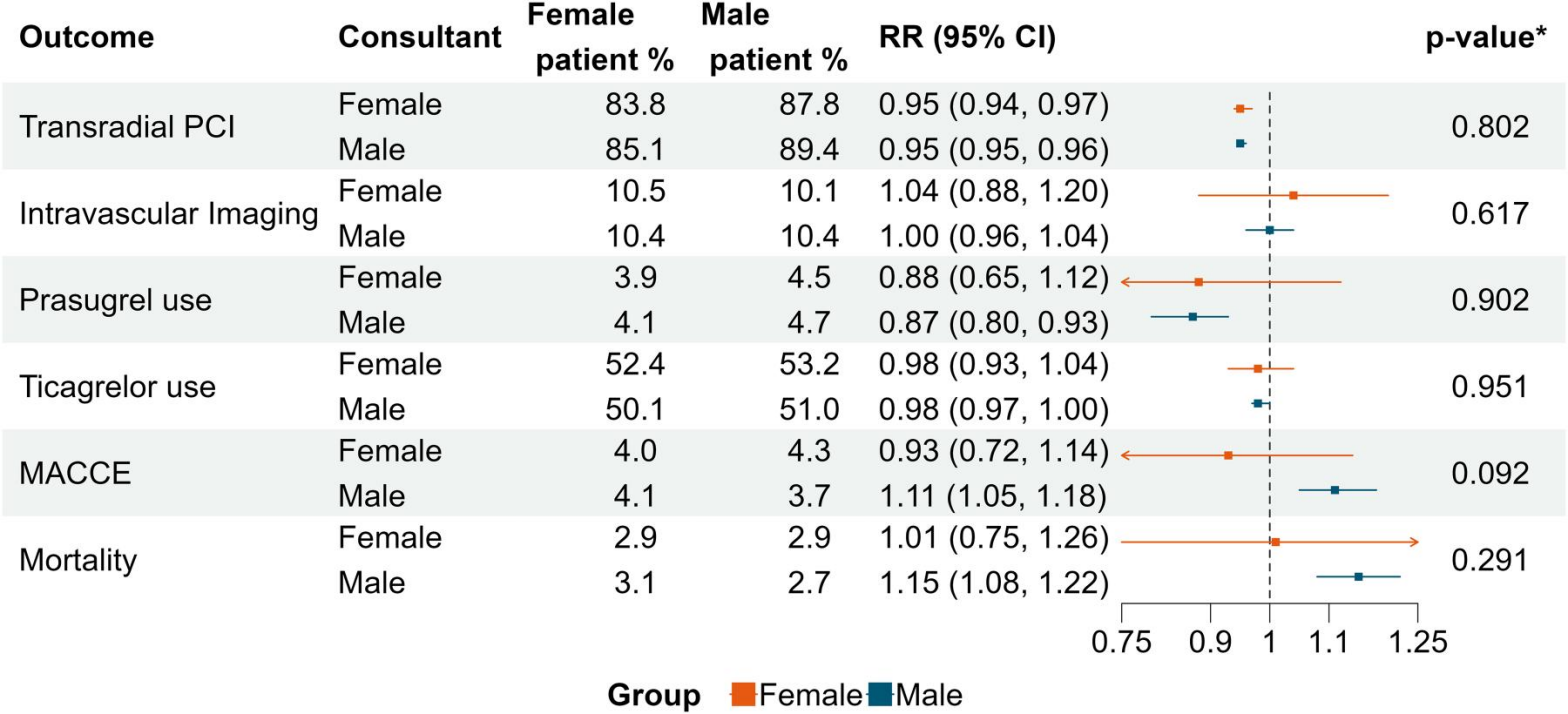
Independent association of patient−operator gender with periprocedural treatments and adverse hospital outcomes. MACCE, Major Adverse Cardiovascular and Cerebrovascular Events; PCI, Percutaneous Coronary Intervention. Estimates extracted following adjustment for covariates; ref.=Male patient. *p‐value tests the interaction between operator and patient gender. [Color figure can be viewed at wileyonlinelibrary.com]

**Table 3:** Independent association of sex concordance/discordance between operator and patient with periprocedural and adverse hospital outcomes.

	Sex discordance % (*N* = 40,370)	Sex concordance % (*N* = 97,742)	RR (95% CI)	*p* value
Transradial PCI	85.5	89.2	0.96 (0.95, 0.96)	<0.001
Intravascular imaging	10.3	10.4	0.99 (0.96, 1.02)	0.586
Prasugrel use	4.1	4.7	0.88 (0.82, 0.94)	<0.001
Ticagrelor use	50.5	51.0	0.99 (0.98, 1.00)	0.123
MACCE	4.1	3.7	1.12 (1.06, 1.18)	<0.001
Mortality	3.1	2.7	1.14 (1.07, 1.21)	<0.001

Abbreviations: MACCE, major adverse cardiovascular and cerebrovascular events; PCI, percutaneous coronary intervention.

Estimates extracted following adjustment for covariates; ref. = sex concordance.

Additionally, following adjustment for covariates, female patients had a 10% higher risk of MACCE than male patients (RR: 1.10 [95% CI: 1.04, 1.16]) (Table 2). This excess risk was particularly evident for procedures performed by male operators, where the risk of MACCE was higher in female than in male patients (adjusted risk: 4.1% vs. 3.7%; RR: 1.11 [95% CI: 1.05, 1.18]) (Figure 2). Conversely, for procedures performed by female operators, the risk of MACCE was higher for male patients than female patients (adjusted risk: 4.3% vs. 4.0%), however corresponding to a non‐significant statistical difference (RR:0.93 [95% CI: 0.72, 1.14]). A direct comparison of these two relative risks indicated a marginal difference between them (*p* = 0.092). Additionally, patient−operator sex discordance was associated with a 12% higher risk of MACCE compared with sex concordance (RR: 1.12 [95% CI: 1.06, 1.18]) (Table 3).

On mortality, following adjustment for covariates, female patients had a 14% higher risk of in‐hospital mortality than male patients (RR:1.14 [95% CI: 1.07, 1.21]) (Table 2). As above, this finding was more profound for PCIs performed by male operators, and no evidence of sex‐based outcome difference was found for PCIs performed by female operators. Specifically, for PCIs performed by male operators, the risk of in‐hospital mortality was 3.1% for female versus 2.7% for male patients (RR: 1.15 [95% CI: 1.08, 1.22]) (Figure 2), whereas for procedures performed by female operators, the risk did not differ according to patient sex. In‐hospital mortality risk also differed according to sex concordance: operator−patient sex discordance was associated with a 14% higher risk compared with sex concordance (RR: 1.14 [95% CI: 1.07, 1.21]) (Table 3).

The STEMI subgroup included 54380 (39.4%) PCI procedures (Supporting Information S1: Table 2). Similarly to above, following adjustment for confounders, female patients were less likely to receive transradial PCI and prasugrel, and were also‐marginally‐less likely to receive intravascular imaging (adjusted risk: 6.2% vs. 6.6%; RR:0.93 [95% CI: 0.85, 1.00]) (Supporting Information S1: Table 3). For prasugrel use and intravascular imaging, the difference was most profoundly indicated for PCI operated by males (Supporting Information S1: Table 4). For STEMI patients, there was no evidence of statistically significant difference in ticagrelor use and MACCE rates by patient sex, whereas risk of in‐hospital mortality was higher for female than male patients (adjusted risk: 5.7% vs. 5.3%; RR:1.08 [95% CI: 1.00, 1.15]; Supporting Information S1: Table 3) and that was majorly evident in procedures by male operators (Supporting Information S1: Table 4). Operator−patient sex discordance for patients with STEMI was associated with less transradial PCI, intravascular imaging, and prasugrel use. Sex discordance was also associated with higher risk of MACCE (RR: 1.07 [95% CI: 1.00, 1.14]) and in‐hospital mortality (RR: 1.08 [95% CI: 1.01, 1.15]) compared with sex concordance (Supporting Information S1: Table 5).

## 4 Discussion

This study evaluated the association between operator–patient sex and periprocedural care and outcomes following PCI in ACS. We found that female patients were less likely to receive guideline‐recommended treatments, including transradial PCI and potent P2Y12 inhibitors, and experienced higher risks of in‐hospital mortality and MACCE compared with male patients. Notably, female patients treated by male operators had worse outcomes, whereas no clear sex‐based differences were observed among female operators, although estimates were imprecise due to the small number of procedures performed by female operators. Additionally, operator–patient sex discordance was associated with lower use of transradial access and prasugrel, and higher risk of adverse outcomes. Central Illustration 1.

**Central Illustration 1:**
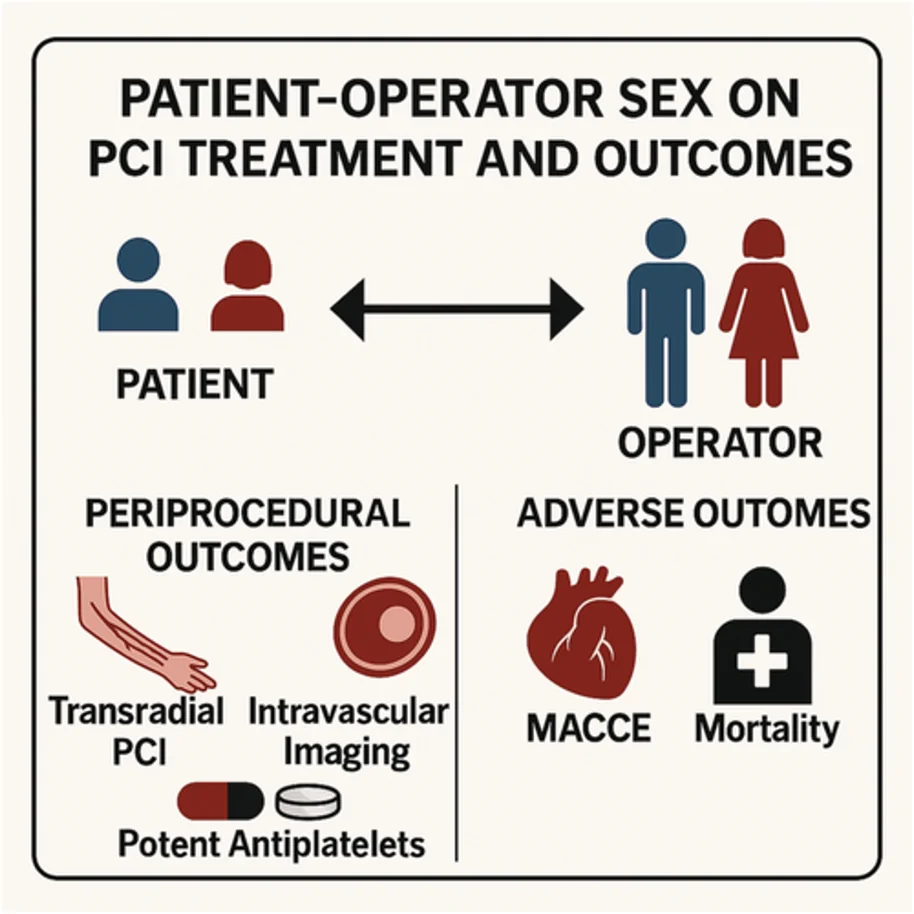
[Color figure can be viewed at wileyonlinelibrary.com

Our study extends the work of Yelavarthy et al. [12] and Dziewiers et al. [15] by incorporating more recent, nationally representative data and evaluating key quality indicators of PCI. This is the first study to evaluate the impact of operator−patient sex on PCI quality metrics like transradial access, intravascular imaging and potent antiplatelets.

Our findings are consistent with previous studies reporting lower use of transradial access and potent antiplatelet therapy in women, as well as worse clinical outcomes [5, 6, 23, 24, 25, 26, 27, 28, 29, 30]. These differences have been attributed to anatomical factors, such as smaller radial arteries and higher susceptibility to vasospasm, as well as concerns regarding bleeding risk and the underrepresentation of women in clinical trials [7, 8, 23, 24, 25, 26, 31, 32]. Despite adjustment for baseline characteristics, sex‐based differences in outcomes persisted, suggesting that disparities in care delivery and treatment decisions may contribute to these findings [28, 30, 33, 34, 35].

This study extends prior work by demonstrating that operator–patient sex interactions may influence both treatment patterns and clinical outcomes. While previous PCI studies did not identify significant associations between operator sex and patient outcomes [12, 15], these were limited by geography or patient population. In contrast, our findings align with broader evidence from medical and surgical settings showing that sex discordance between patients and physicians may be associated with worse outcomes, particularly among female patients [9, 10, 11, 16, 17]. For example, Greenwood et al. [16] reported higher mortality among female patients treated by male physicians following acute myocardial infarction.

Several mechanisms may explain these findings. Differences in the use of guideline‐recommended therapies are likely to play an important role, as adherence to evidence‐based care has been consistently associated with improved outcomes [24, 36, 37, 38, 39]. In addition, patient−operator interactions, including communication and potential implicit bias, may influence clinical decision‐making and treatment implementation [40]. The lower use of transradial access and prasugrel in discordant operator–patient pairs suggests that interpersonal or perceptual factors may influence the application of guideline‐recommended strategies, whereas treatments guided more strictly by protocols, such as intravascular imaging or ticagrelor use, showed less variation.

These findings have important implications for clinical practice and workforce planning. Improving adherence to guideline‐recommended treatments through standardized protocols may help reduce sex‐based disparities in outcomes. The association between sex discordance and adverse outcomes highlights the need to address both interpersonal and structural factors influencing care delivery. Notably, the absence of adverse outcome differences among female operators, despite limited sample size, supports efforts to increase diversity within the interventional cardiology workforce. Greater representation may contribute to more equitable care and improved patient outcomes. Future research should further investigate the mechanisms underlying these associations and evaluate interventions aimed at improving communication, reducing bias, and ensuring consistent delivery of evidence‐based care. Finally, these findings are descriptive and should not be interpreted as causal, while they call for further research into the mechanisms driving these associations and for interventions aimed at enhancing communication, mitigating bias, and promoting equity in patient care. A target trial emulation engaging a larger sample to reduce uncertainty or a bigger window in the outcomes, such as 30‐day or 1‐year mortality, may explain underlying mechanisms.

The main strength of this study is the inclusion of real‐world, unselected patients from a national PCI registry, with a large sample size that enhances generalizability to a broader PCI population. However, there are some limitations. First, operator sex was obtained through online searches, subject to inaccuracies liable to misclassification bias. Nonetheless, these classifications were cross‐validated with expert knowledge within our research team, including the national audit lead responsible for individual operator PCI reporting, thereby minimizing potential bias. Second, the BCIS dataset does not capture data for patients managed medically. Third, information on hospital characteristics such as size, teaching status and data on operator demographics such as age and years of experience are not captured in this registry and therefore were not accounted for in the models. Fourth, procedures may involve both consultants and fellows with varying levels of supervision and hands‐on participation. As operator‐level reporting in the registry is attributed to the responsible consultant, our analysis reflects consultant sex; however, variation in trainee involvement and experience may introduce heterogeneity in procedural conduct and outcomes, which could influence interpretation of operator‐level effects.

Fifth, our study evaluated sex as a biological variable and was unable to assess patient or physician gender identity, and other intersectional variables such as socioeconomic status and disability. Sixth, the lower proportion of PCIs by female operators resulted in wide confidence intervals and increased uncertainty in some estimates. Lastly, like in all other observational studies, the possibility of residual confounding could not be ruled out, despite adjustment for all known and measurable confounders, therefore, causality could not be inferred.

## 5 Conclusion

Female patients were less likely to receive guideline‐recommended PCI and experienced worse outcomes than male patients. While outcomes appeared less favorable among female patients treated by male operators, this finding should be interpreted cautiously given the imbalance in operator sample size and the modest differences observed, as well as the well‐established sex disparities in PCI outcomes reported in prior studies.

These findings reinforce the importance of ensuring consistent delivery of guideline‐recommended treatments to all patients regardless of sex. Improving adherence to evidence‐based management may help reduce mortality and narrow sex‐based disparities in outcomes. Interventions aimed at standardizing care, improving communication, and addressing potential sources of bias may further support more equitable treatment across operator–patient groups.

## Conflicts of Interest

The authors declare no conflicts of interest.

## Supporting information


Supporting File
