# Supplementary material for: Interaction of Operator−Patient Sex on Peri‐Procedural Treatments and Major Coronary Outcomes After Percutaneous Coronary Intervention
Source: Catheter Cardiovasc Interv. 2026 Aug 5;108(4):1520–8. doi: 10.1002/ccd.70799 (PMC13667334; doi:10.1002/ccd.70799)
Supplement: Supplementary file 1 [file CCD-108-1520-s001.docx]

**SUPPLEMENTARY MATERIAL**

**Figure A.1: Patient inclusion/exclusion criteria**

**Table A.1: Proportions of missing data for covariates**

| **Variable** | **n(%)** |
| --- | --- |
| N | 138112 |
| Ethnicity | 28126 (20.4%) |
| BMI | 19490 (14.1%) |
| Smoking status | 10882 (7.9%) |
| LVEF | 71846 (52.0%) |
| Previous MI | 2006 (1.5%) |
| Previous CABG | 968 (0.7%) |
| Previous PCI | 1467 (1.1%) |
| Hypercholesterolaemia | 2432 (1.8%) |
| Hypertension | 2432 (1.8%) |
| Peripheral vascular disease | 2432 (1.8%) |
| Cerebrovascular event | 2432 (1.8%) |
| Valvular heart disease | 2432 (1.8%) |
| Cardiogenic Shock Pre- Procedure | 980 (0.7%) |
| Renal failure | 4318 (3.1%) |
| Diabetes Mellitus | 2274 (1.6%) |
| Left main disease | 21706 (15.7%) |
| Multivessel disease | 7491 (5.4%) |
| Chronic total occlusion | 4978 (3.6%) |

BMI: Body Mass Index; CABG: Coronary Artery Bypass Grafting;

LVEF: Left Ventricular Ejection Fraction; MI: Myocardial Infarction;

PCI: Percutaneous Coronary Intervention

**Table A.2: Demographics and clinical characteristics tabulated by sex of patients and operators for STEMI patients (n, (%))**

| **Operator Sex** | **Male** | | **Female** | | **Overall** |
| --- | --- | --- | --- | --- | --- |
| **Patient Sex** | **Male** | **Female** | **Male** | **Female** |  |
| N | 38212 | 13002 | 2358 | 808 | 54380 |
| Age, mean (SD) | 62.2 (12.2) | 68.5 (13.0) | 61.8 (12.0) | 67.5 (13.1) | 63.8 (12.7) |
| Ethnicity |  |  |  |  |  |
| White | 25842 (85.3) | 9203 (89.3) | 1583 (80.8) | 601 (87.0) | 37229 (86.1) |
| Not white | 4460 (14.7) | 1102 (10.7) | 377 (19.2) | 90 (13.0) | 6029 (13.9) |
| BMI, mean (SD) | 28.0 (4.9) | 27.8 (6.0) | 28 (4.7) | 28.2 (5.8) | 27.9 (5.2) |
| Smoking |  |  |  |  |  |
| Never smoker | 12552 (36.1) | 5296 (45.0) | 771 (35.7) | 315 (43.6) | 18934 (38.3) |
| Current/Ex smoker | 22263 (63.9) | 6483 (55.0) | 1388 (64.3) | 407 (56.4) | 30541 (61.7) |
| Previous MI |  |  |  |  |  |
| No | 32585 (86.7) | 11490 (89.7) | 1988 (85.4) | 720 (90.5) | 46783 (87.4) |
| Yes | 4986 (13.3) | 1326 (10.3) | 339 (14.6) | 76 (9.5) | 6727 (12.6) |
| Previous CABG |  |  |  |  |  |
| No | 36856 (97.4) | 12700 (98.4) | 2274 (97.7) | 787 (98.5) | 52617 (97.7) |
| Yes | 998 (2.6) | 200 (1.6) | 54 (2.3) | 12 (1.5) | 1264 (2.3) |
| Previous PCI |  |  |  |  |  |
| No | 33051 (87.7) | 11566 (90.1) | 2012 (86.8) | 733 (92.0) | 47362 (88.3) |
| Yes | 4638 (12.3) | 1277 (9.9) | 307 (13.2) | 64 (8.0) | 6286 (11.7) |
| Hypercholesterolaemia |  |  |  |  |  |
| No | 24664 (65.9) | 8303 (65.0) | 1540 (66.6) | 535 (67.0) | 35042 (65.8) |
| Yes | 12742 (34.1) | 4471 (35.0) | 771 (33.4) | 264 (33.0) | 18248 (34.2) |
| Hypertension |  |  |  |  |  |
| No | 21988 (58.8) | 6564 (51.4) | 1337 (57.9) | 437 (54.7) | 30326 (56.9) |
| Yes | 15418 (41.2) | 6210 (48.6) | 974 (42.1) | 362 (45.3) | 22964 (43.1) |
| PVD |  |  |  |  |  |
| No | 36374 (97.2) | 12410 (97.2) | 2245 (97.1) | 774 (96.9) | 51803 (97.2) |
| Yes | 1032 (2.8) | 364 (2.8) | 66 (2.9) | 25 (3.1) | 1487 (2.8) |
| Previous stroke |  |  |  |  |  |
| No | 36223 (96.8) | 12238 (95.8) | 2227 (96.4) | 761 (95.2) | 51449 (96.5) |
| Yes | 1183 (3.2) | 536 (4.2) | 84 (3.6) | 38 (4.8) | 1841 (3.5) |
| Valvular heart disease |  |  |  |  |  |
| No | 36979 (98.9) | 12546 (98.2) | 2269 (98.2) | 783 (98.0) | 52577 (98.7) |
| Yes | 427 (1.1) | 228 (1.8) | 42 (1.8) | 16 (2.0) | 713 (1.3) |
| LVEF |  |  |  |  |  |
| Good | 5715 (38.5) | 2036 (41.0) | 372 (38.8) | 122 (38.1) | 8245 (39.1) |
| Moderate/Poor | 9128 (61.5) | 2928 (59.0) | 587 (61.2) | 198 (61.9) | 12841 (60.9) |
| Diabetes mellitus |  |  |  |  |  |
| No | 30695 (82.2) | 10066 (79.1) | 1881 (81.7) | 637 (80.2) | 43279 (81.4) |
| Yes | 6643 (17.8) | 2664 (20.9) | 421 (18.3) | 157 (19.8) | 9885 (18.6) |
| Kidney disease |  |  |  |  |  |
| No | 35470 (98.3) | 12024 (98.0) | 2232 (98.3) | 763 (98.8) | 50489 (98.3) |
| Yes | 603 (1.7) | 243 (2.0) | 38 (1.7) | 9 (1.2) | 893 (1.7) |
| Cardiogenic shock |  |  |  |  |  |
| No | 35049 (92.2) | 11778 (91.0) | 2186 (93.5) | 730 (90.9) | 49743 (92.0) |
| Yes | 2955 (7.8) | 1170 (9.0) | 153 (6.5) | 73 (9.1) | 4351 (8.0) |
| MVL Disease |  |  |  |  |  |
| No | 25030 (69.4) | 8954 (72.9) | 1619 (73.3) | 565 (75.7) | 36168 (70.5) |
| Yes | 11013 (30.6) | 3325 (27.1) | 590 (26.7) | 181 (24.3) | 15109 (29.5) |
| Left Main disease |  |  |  |  |  |
| No | 30648 (97.0) | 10492 (97.5) | 1713 (96.2) | 594 (96.9) | 43447 (97.1) |
| Yes | 960 (3.0) | 268 (2.5) | 67 (3.8) | 19 (3.1) | 1314 (2.9) |
| CTO |  |  |  |  |  |
| No | 36221 (97.7) | 12413 (98.2) | 2212 (96.6) | 767 (98.1) | 51613 (97.7) |
| Yes | 870 (2.3) | 233 (1.8) | 77 (3.4) | 15 (1.9) | 1195 (2.3) |

BMI: Body Mass Index; CABG: Coronary Artery Bypass Grafting; CTO: Chronic Total Occlusion; LVEF: Left Ventricular Ejection Fraction; MI: Myocardial Infarction; MVL: Multivessel; PCI: Percutaneous Coronary Intervention; PVD: Peripheral Vascular Disease

**Table A.3: Independent association of patient’s gender with periprocedural treatments and adverse hospital outcomes for STEMI patients**

|  | **Female patient % (N=13,810)** | **Male patient % (N=40,570)** | **RR (95% CI)** | **p-value** |
| --- | --- | --- | --- | --- |
| Transradial PCI | 84.9 | 88.9 | 0.96 (0.95,0.96) | <0.001 |
| Intravascular Imaging | 6.2 | 6.6 | 0.93 (0.85,1.00) | 0.060 |
| Prasugrel use | 8.1 | 9.0 | 0.90 (0.84,0.97) | 0.004 |
| Ticagrelor use | 60.2 | 60.4 | 1.00 (0.98,1.02) | 0.723 |
| MACCE | 6.9 | 6.5 | 1.06 (0.99,1.12) | 0.115 |
| Mortality | 5.7 | 5.3 | 1.08 (1.00,1.15) | 0.045 |

*Estimates extracted following adjustment for covariates; ref.=Male patient

MACCE: Major Adverse Cardiovascular and Cerebrovascular Events; PCI: Percutaneous Coronary Intervention

**Table A.4: Independent association of patient - operator gender with periprocedural treatments and adverse hospital outcomes, for STEMI patients**

|  | **Operator** | **Female patient %** | **Male patient %** | **RR (95% CI)** | **p-value**** |
| --- | --- | --- | --- | --- | --- |
| Transradial PCI | Female  Male | 82.5  85.1 | 85.8  89.0 | 0.96 (0.93,0.99)  0.96 (0.95,0.96) | 0.715 |
| Intravascular Imaging | Female  Male | 7.0  6.1 | 6.5  6.6 | 1.08 (0.75,1.42)  0.92 (0.84,0.99) | 0.338 |
| Prasugrel use | Female  Male | 7.5  8.1 | 8.6  9.0 | 0.87 (0.61,1.13)  0.90 (0.84,0.97) | 0.806 |
| Ticagrelor use | Female  Male | 64.3  59.9 | 65.2  60.1 | 0.99 (0.92,1.06)  1.00 (0.98,1.02) | 0.772 |
| MACCE | Female  Male | 6.7  6.9 | 7.0  6.5 | 0.96 (0.71,1.21)  1.06 (0.99,1.13) | 0.441 |
| Mortality | Female  Male | 5.3  5.7 | 5.4  5.3 | 0.99 (0.71,1.28)  1.08 (1.00,1.16) | 0.543 |

* Estimates extracted following adjustment for covariates; ref.=Male patient

**p-value tests the interaction between operator and patient gender.

MACCE: Major Adverse Cardiovascular and Cerebrovascular Events; PCI: Percutaneous Coronary Intervention

**Table A.5: Independent association of sex concordance/ discordance between operator and patient with periprocedural and adverse hospital outcomes for STEMI patients**

|  | **Sex discordance % (N=15,360)** | **Sex concordance % (N=39,020)** | **RR (95% CI)** | **p-value** |
| --- | --- | --- | --- | --- |
| Transradial PCI | 85.2 | 88.9 | 0.96 (0.95,0.97) | <0.001 |
| Intravascular Imaging | 6.1 | 6.7 | 0.92 (0.85,0.99) | 0.021 |
| Prasugrel use | 8.2 | 9.0 | 0.92 (0.85,0.98) | 0.009 |
| Ticagrelor use | 60.7 | 60.2 | 1.01 (0.99,1.03) | 0.389 |
| MACCE | 6.9 | 6.5 | 1.07 (1.00,1.14) | 0.047 |
| Mortality | 5.7 | 5.3 | 1.08 (1.01,1.15) | 0.032 |

*Estimates extracted following adjustment for covariates; ref.=sex concordance

MACCE: Major Adverse Cardiovascular and Cerebrovascular Events; PCI: Percutaneous Coronary Intervention
